# Lipid peroxidation and apoptotic response in rat brain areas induced by long‐term administration of nandrolone: the mutual crosstalk between ROS and NF‐kB

**DOI:** 10.1111/jcmm.12748

**Published:** 2016-02-01

**Authors:** Emanuela Turillazzi, Margherita Neri, Daniela Cerretani, Santina Cantatore, Paola Frati, Laura Moltoni, Francesco Paolo Busardò, Cristoforo Pomara, Irene Riezzo, Vittorio Fineschi

**Affiliations:** ^1^ Institute of Legal Medicine Department of Clinical and Experimental Medicine University of Foggia Foggia Italy; ^2^ Pharmacology Unit Department of Medicine Surgery and Neuroscience University of Siena Italy; ^3^ Department of Anatomical, Histological, Forensic and Orthopaedic Sciences Sapienza University of Rome Roma Italy; ^4^ Neuromed Istituto Mediterraneo Neurologico (IRCCS) Pozzilli Isernia Italy

**Keywords:** nandrolone decanoate, oxidative stress, neurotoxicity, apoptosis

## Abstract

The aim of this study was to evaluate the played by oxidative stress in the apoptotic response in different brain areas of rats chronically treated with supra‐physiological doses of nandrolone decanoate (ND). Immunohistochemical study and Western blot analysis were performed to evaluate cells' apoptosis and to measure the effects of expression of specific mediators, such as NF‐κB (nuclear factor kappa‐light‐chain‐enhancer of activated B cells), Bcl‐2 (B‐cell lymphoma 2), SMAC/DIABLO (second mitochondria‐derived activator of caspases/direct IAP‐binding protein with low PI) and VMAT2 (vesicular monoamine transporter 2) on apoptosis. The results of the present study indicate that a long‐term administration of ND promotes oxidative injury in rat brain specific areas. A link between oxidative stress and NF‐κB signalling pathways is supported by our results. In addition to high levels of oxidative stress, we consistently observed a strong immunopositivity to NF‐κB. It has been argued that one of the pathways leading to the activation of NF‐κB could be under reactive oxygen species (ROS)‐mediated control. In fact, growing evidence suggests that although in limited doses, endogenous ROS may play an activating role in NF‐κB signalling, while above a certain threshold, they may negatively impact upon this signalling. However, a mutual crosstalk between ROS and NF‐κB exists and recent studies have shown that ROS activity is subject to negative feedback regulation by NF‐κB, and that this negative regulation of ROS is the means through which NF‐κB counters programmed cells.

## Introduction

Anabolic androgenic steroids (AASs) are a group of synthetic compounds obtained by selective chemical manipulations of the 19‐carbon testosterone molecule that affect the pharmacokinetics as well as the ratio of the anabolic/androgenic effect 1. Misuse of AASs by athletes is widely acknowledged, and worldwide non‐medical use is increasing in adolescents and adults, typically in individuals seeking physical strength, enhanced appearance and performance 2, 3, 4, 5, 6, 7, 8. AASs can be legally prescribed to treat conditions resulting from steroid hormone deficiency, such as delayed puberty and hypogonadism, as well as other diseases, such as bone marrow failure syndromes, bone mineralization and some muscle‐wasting disorders 9.

Although the potential effects on the nervous system have not been well defined, a wide range of physical and psychiatric adverse effects has been described in the literature 10, 11. Early behavioural effects include increased confidence, energy and motivation accompanied by irritability and agitation 12, 13, whereas prolonged use is usually associated with loss of inhibition and impulsive and markedly aggressive behaviour 13 by significantly modifying both serotonergic and noradrenergic neurotransmission 14. Currently, it is not yet fully clarified whether AASs are toxic to neurons and whether their abuse is a risk factor for chronic neurodegenerative disorders, although growing evidence supports a neurodegenerative potential for AASs 15, 16. The neurodegenerative effects of long‐term AASs abuse seem to be a phenomenon that has not yet been taken into consideration, probably because of the fact that most of the AAS users are still under the age of 50 and even if they might have incurred in neurotoxic effects, they are still too young to exhibit gross cognitive or motor deficits 17, even though neuronal loss has been observed on human AASs abusers 18.

Although the origin of AASs neurodegeneration might be multi‐factorial, oxidative stress could play a critical role. In fact, oxidative stress has been involved in many neurodegenerative human diseases, such as Alzheimer's disease, Parkinson's disease (PD), Huntington's disease, amyotrophic lateral sclerosis and HIV‐associated neurocognitive disorder 19, 20, 21, 22, 23, and the potential effects of disrupting the redox signalling of AASs is evident and this kind of toxicity occurs in numerous organs and systems 24. In addition, recent animal studies have shown that increased neuronal susceptibility to apoptotic stimuli could explain the neurotoxic effects of AASs 25. Long‐term administration of certain AASs leads to behavioural changes in the central nervous system in rodents 26, 27, 28, 29, 30, 31, 32, which may underlie some of the behavioural changes that are observed in AASs abusers 33.

As there is growing evidence of the potential role of oxidative stress and apoptosis for AASs‐mediated neurotoxicity, the aim of this study was to evaluate the role played by oxidative stress in the apoptotic response in different brain areas of rats chronically treated with supra‐physiological doses of nandrolone decanoate (ND), one of the most frequently abused AASs.

## Materials and methods

The experiments were performed on 40 adult male Wistar rats (Wistar, Charles River, Lecco, Italy) weighing 200–250 g (10 weeks old). All experimental procedures were in compliance with the EEC Directive (86/609/EEC) on the protection of animals used for experimental and other scientific purposes, and were approved by the Ethical Committee for the Use of Laboratory Animals of the University of Siena. All efforts were made to minimize animal suffering and to reduce the number of animals used.

At the beginning of the experiments there was no statistically significant difference in animals' bodyweight within the group (*P* > 0.20), as well as between the groups (*P* > 0.30). All animals were housed in four per cage (55 × 35 × 30 cm), under standard conditions (23 ± 2°C, 50–60% relative humidity, 12 hr/12 hr light/dark cycle with lights on at 08:00, and with free access to food and water). The animals were randomly divided into two groups A: ND treated group and B: control group (submitted to vehicle injection; peanut oil with 10% of benzoic alcohol). Steroid and vehicle were administered by a single intramuscular injection twice a week for 8 weeks. The rats of group A (20 animals) received 3.75 mg ND/kg/week (1.875 mg/kg twice per week). The rats of group B (20 animals) received vehicle, twice a week. One week after the last injection, the rats were killed by decapitation, and blood was immediately collected. The brains were excised and were placed dorsal side up in an ice‐chilled rat brain matrix (World Precision Instruments, Inc., Aston, Stevenage, UK) with slits spaced at 1 mm. Using an ice‐chilled razor blade, the target regions were dissected according to the atlas of Paxinos and Watson. In each case, samples from (pre) frontal cortex (PFC), striatum (S), hippocampus (Hipp) and cerebellum (Cer) were taken. A portion of each sample was immediately frozen in liquid nitrogen and stored at −80°C. The remaining samples were fixed in 10% buffered formalin for 48 hrs.

### Biochemical analysis

#### Malondialdehyde assessment

The extent of lipid peroxidation, a marker of oxidative stress, in rat brain areas was estimated using malondialdehyde (MDA) level calculation. Samples of brain areas were homogenized in a 0.04 M K^+^‐phosphate buffer (pH 7.4) containing 0.01% butyl hydroxytoluene (1:5 w/v, 0°C) to prevent the artificial oxidation of polyunsaturated free fatty acids during the assay. This homogenate was deproteinized with acetonitrile (1:1) and then centrifuged at 3000 × g for 15 min. The supernatants were used for MDA‐analysis after pre‐column derivatization with 2,4‐dinitrophenylhydrazine. The MDA‐hydrazone was quantified by isocratic reversed‐phase high‐performance liquid cromatography (HPLC) method with UV detection as described by Shara *et al*. 34.

### Histopathological study

Paraffin‐embedded brain tissue specimens were sectioned at 4 μm and stained with haematoxylin and eosin. In addition, an immunohistochemical investigation was performed with antibodies anti‐NF‐κB (nuclear factor kappa‐light‐chain‐enhancer of activated B cells), Bcl‐2 (B‐cell lymphoma 2), SMAC/DIABLO (second mitochondria‐derived activator of caspases/direct IAP‐binding protein with low PI), VMAT‐2 (vesicular monoamine transporter 2) and apoptosis with TUNEL assay. We used 3‐μm‐thick paraffin sections mounted on slides covered with 3, amminopropyl‐triethoxysilane (Fluka, Buchs, Switzerland). A pre‐treatment was necessary to facilitate antigen retrieval and to increase membrane permeability to the antibodies: for NF‐κB (Santa Cruz Biotechnology, Santa Cruz, CA, USA), boiling 0.25 M ethylenediaminetetraacetic acid buffer; for Bcl‐2 (Millipore–Upstate, Temecula, CA, USA), SMAC/DIABLO (Millipore–Chemicon) boiling in 0.1 M citric acid buffer, and for antibody anti‐VMAT2 (Chemicon), 5 min. proteolytic enzyme at 20°C (Dako, Copenhagen, Denmark). For TUNEL assay (Millipore–Chemicon), we used TdT enzyme: the sections were immerged in proteinase K (20 μg/ml of TRIS) for 15 min. at 20°C. The primary antibody was applied in a 1:50 ratio for NF‐κB and Bcl‐2. The incubation of the primary antibody was for 120 min. at 20°C. For TUNEL assay the sections were covered with the TdT enzyme, diluted in a ratio of 30% in reaction buffer (Apotag Plus Peroxidase In Situ Apoptosis Detection Kit; Chemicon) and incubated for 60 min. at 38°C. The detection system utilized was the LSAB+ kit (Dako), a refined avidin–biotin technique in which a biotinylated secondary antibody reacts with several peroxidase‐conjugated streptavidin molecules. The positive reaction was visualized with 3,3‐diaminobenzidine peroxidation, according to standard methods. Then, the sections were counterstained with Mayer's Haematoxylin, dehydrated, cover‐slipped and observed under a Leica DM4000B optical microscope (Leica, Cambridge, UK). The samples were also examined under a confocal microscope, and a three‐dimensional reconstruction was performed (True Confocal Scanner; Leica TCS SPE). For semiquantitative analysis, slides were scored in a blinded manner by two observers. Staining pattern within each sample was assessed semiquantitatively in the scale 0–5 as follows: −: no immunoreactivity (0%); +: mild immunopositivity in scattered cells (10%); ++: immunopositivity in up to one‐third of cells (33%); +++: immunopositivity in up to one half of cells (50%) and ++++: strong immunopositivity in the majority or in all cells (100%).

### Western blot analysis

Western blot analysis was performed. Approximately, 100 mg of frozen brain tissue was dissected and immediately transferred to the RIPA buffer with a protease inhibitor cocktail, and homogenized on ice, utilizing the homogenizer Silent Crusher. The homogenate was centrifuged (12,000 × g for 10 min. at 4°C). The supernatant was collected, estimated by Qubit Fluorometer (Invitrogen, Thermo Fisher Scientific, Waltham, MA, USA), and boiled for 5 min., at 95°C. Brain total protein extracts (approximately 40 μg/lane) were run on 4–15% SDS PAGE at 80 V for about 2.5 hrs. For Western blot analysis, proteins from SDS gels were electrophoretically transferred to nitrocellulose membranes in mini trans blot apparatus (1 hr at 250 mA). Non‐specific binding was blocked by incubating membranes in Western blocker solution for 1 hr at room temperature. The membranes were incubated with primary antibodies anti‐diluted in Western blocker solution, in a 1:400 ratio overnight at 4°C. Blots were washed with PBS (Tween‐20) and then incubated for 1 hr at room temperature with HRP (horseradish peroxidase)‐conjugated secondary antibodies diluted in Western blocker solution, in a 1:2000 ratio. Membranes were washed with PBS/Tween‐20, and the immune reaction was developed in IMMUNOSTAR Kit Western C (Bio‐Rad Laboratories, Segrate, Milan, Italy) and then visualized by chemiluminescent detection methods. The light was then detected by a photographic film. The image was analysed by Versadoc (Bio‐Rad Laboratories), which detected the chemiluminescent blots of protein staining.

### Statistical analysis

Values are presented as mean S.D. The unpaired two‐way Student's *t*‐test was used to compare the results obtained for ND treated rat group with the control group. *P* < 0.05 was accepted as indicative of a significant difference between the two groups.

## Results

### MDA evaluation

**Figure 1:**
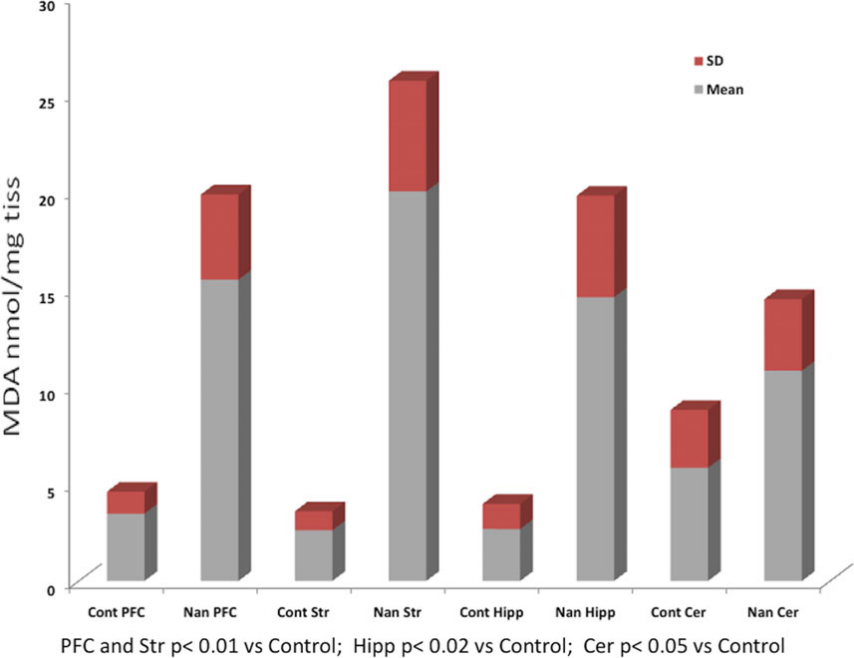
MDA (nmol/mg tissue) in rat brain areas after administration of nandrolone decanoate 1.875 mg/kg twice per week by intramuscular injection for 8 weeks (each value is the mean ± S.D. of three animals). Cont: Control; PFC: frontal cotex; Str: striatum; Hipp: hippocampus; Cer: cerebellum.

The MDA levels were used as a lipoperoxidation index and evidence of oxidative damage. The results obtained showed a strong and significant increase in MDA concentrations (Table 1) in all brain areas examined respect to controls: + 347% PFC, + 669% S, + 446% Hipp, + 86% Cer (Fig. 1).

**Table 1:** MDA (nmol/mg tissue) in rat brain areas after administration of nandrolone decanoate 1.875 mg/kg twice per week by intramuscular injection for 8 weeks (each value is the mean ± S.D. of three animals)

	Cont PFC	Nan PFC	Cont Str	Nan Str	Cont Hipp	Nan Hipp	Cont Cer	Nan Cer
Mean	3.5	15.5	2.61	20.1	2.7	14.6	5.8	10.8
S.D.	1.13	4.38	0.98	5.69	1.29	5.21	2.98	3.7

PFC and Str *P* < 0.01 *versus* Cont; Hipp *P* < 0.02 *versus* Cont; Cer *P* < 0.05 *versus* Cont.

Cont: Control; Nan: nandrolone; PFC: frontal cotex; Str: striatum; Hipp: hippocampus; Cer: cerebellum.

### Histopathological results

The microscopic evaluation of the sections stained with haematoxylin and eosin revealed in the treated group: red neurons, nuclear shrinkage and perivascular haemorrhages.

The immunohistochemical study of the samples, for each antibody revealed the immunohistochemical findings and gradation of the immunohistochemical reaction were described with an ordinal scale and the median value was reported (Table 2).

**Table 2:** Responses NF‐κB, Bcl‐2, VMAT2 and apoptosis with TUNEL assay in brain specimens

	Cont PFC	Nan PFC	Cont Str	Nan Str	Cont Hipp	Nan Hipp	Cont Cer	Nan Cer	Statistical value Nan *versus* Cont
Anti‐NF‐κB	+	+++	+	+++	+	+++	+	+	***
Anti‐Bcl‐2	+	+++	+	+++	+	+++	+	+	***
TUNEL assay	+	+++	+	+++	+	+++	+	+	***
Anti‐VMAT2	+++	+	+++	+	+++	+	+	+	***
SMAC/DIABLO	+	+++	+	+++	+	+++	+	+	***

NS: *P* > 0.05; *: *P* < 0.05; **: *P* < 0.01; ***: *P* < 0.001. Intensity of immunopositivity was assessed semiquantitatively in the scale 0–5 as follows: −: no immunoreactivity (0%); +: mild immunopositivity in scattered cells (10%); ++: immunopositivity in up to one‐third of cells (33%); +++: immunopositivity in up to two‐third of cells (70%) and ++++: strong immunopositivity in the majority or all cells (100%). In cases of divergent scoring, a third observer decided the final category.

Cont: Control; Nan: nandrolone; PFC: frontal cortex; Str: striatum; Hipp: hippocampus; Cer: cerebellum.

*, **, *** is the value of P. It is an international standard.

+, ++, +++, ++++ is the value of semiquantitative analysis (see the text)

#### NF‐κB

**Figure 2:**
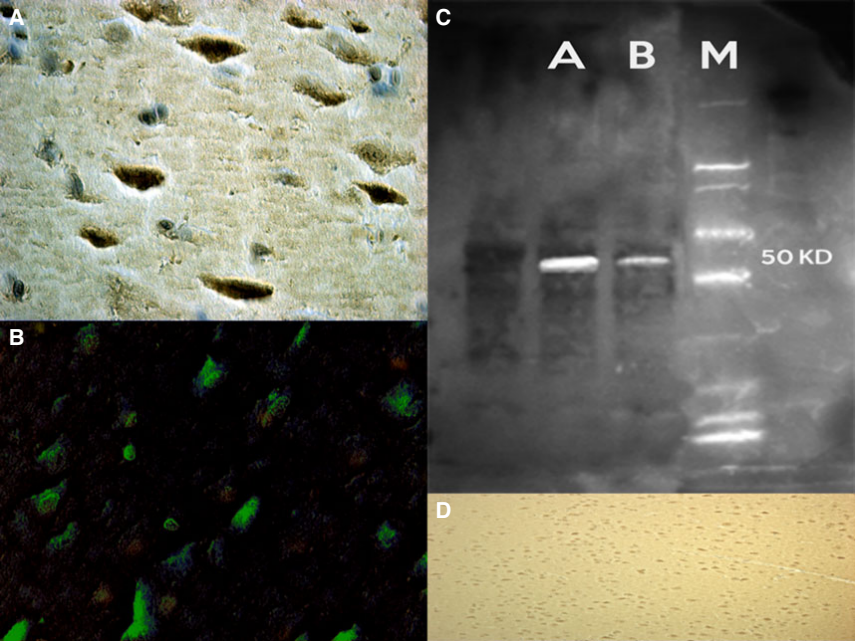
Strong and uniform NF‐kB neuronal positivity was found in the frontal cortex (**A**), striatum and hippocampus (**B**), of the nandrolone group. Western blot analysis detects the chemiluminescent blots of NF‐kB (**C**). (**D**) control group with negative results.

Anti‐NF‐κB provided strong neuronal positive reaction in brain samples of the treated rats compared to the control group, particularly in PFC, S and Hipp samples. Cerebellum areas had a weaker positivity (Fig. 2).

#### Bcl‐2

**Figure 3:**
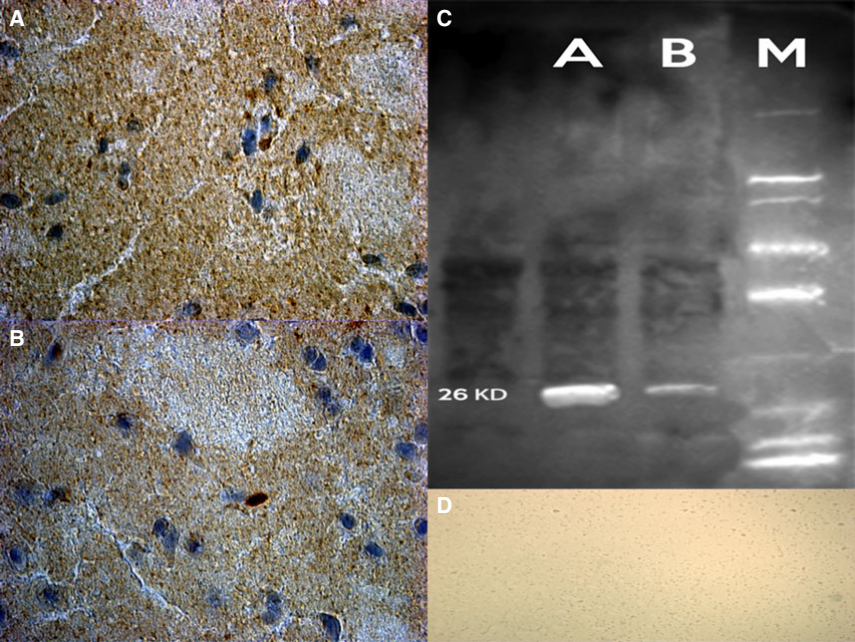
Confocal laser scanning microscopy showed markedly Bcl‐2 positive cytoplasmic reaction (in brown) on the striatum (**A**) and hippocampus (in brown) (**B**) in rats after nandrolone treatment. (**C**) Western blot analysis detects the chemiluminescent blots of Bcl‐2 in the treated group. (**D**) control group with negative results.

We found a strong positive reaction to the Bcl‐2 in ND group compared to control rats. In detail, our findings revealed that PFC, S and Hipp samples had a stronger positive reaction, whereas a weaker positive reaction was detected in cerebellum (Fig. 3).

#### SMAC/DIABLO

**Figure 4:**
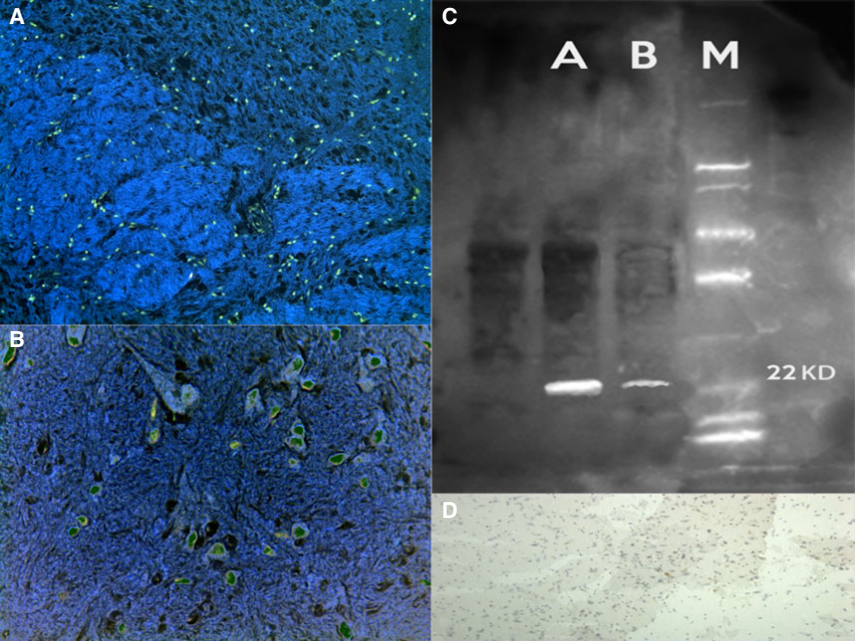
A strong SMAC/DIABLO localization on the dendrites and neuronal cell body positivity located in the frontal cortex (**A**), striatum (**B**) and hippocampus areas was revealed for treated rats. (**C**) Chemiluminescent blots of SMAC/DIABLO in the treated group. (**D**) Control group with negative results.

A strong localization on the dendrites and neuronal cell body positivity located in PFC, S and Hipp areas was revealed for treated rats, compared to control group (Fig. 4).

#### VMAT‐2

**Figure 5:**
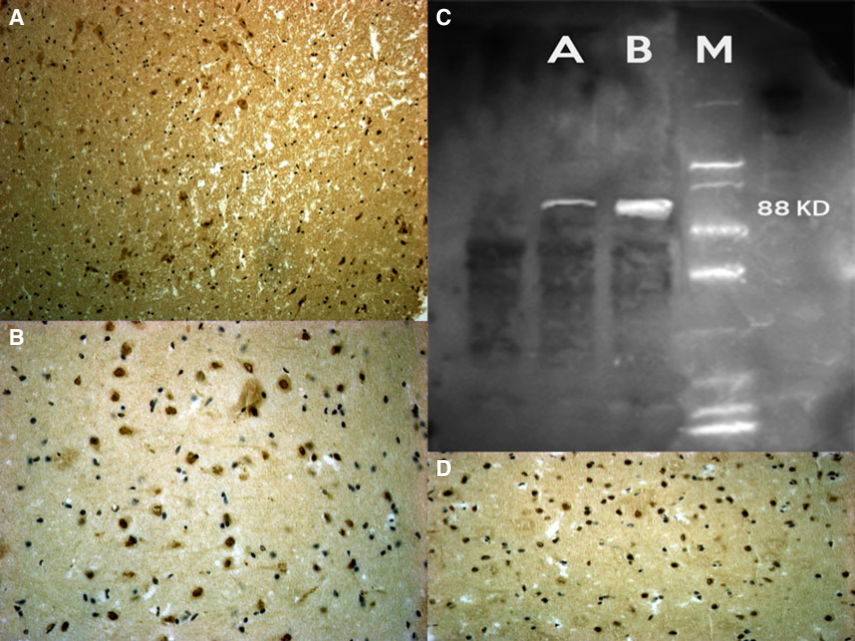
VMAT‐2 weaker reactions on the dendrites and neuronal cell bodies of treated rats compared to controls (**D**). In particular the most significant difference was found in striatum (**A**) and hippocampus (**B**) samples. (**C**) Western blot analysis detects the chemiluminescent blots of VMAT2 in the treated group.

Anti‐VMAT‐2 immunopositivity was significantly weaker on the dendrites and neuronal cell bodies of treated rats compared to controls. In particular the most significant difference was found in S and Hipp samples (Fig. 5).

#### TUNEL

**Figure 6:**
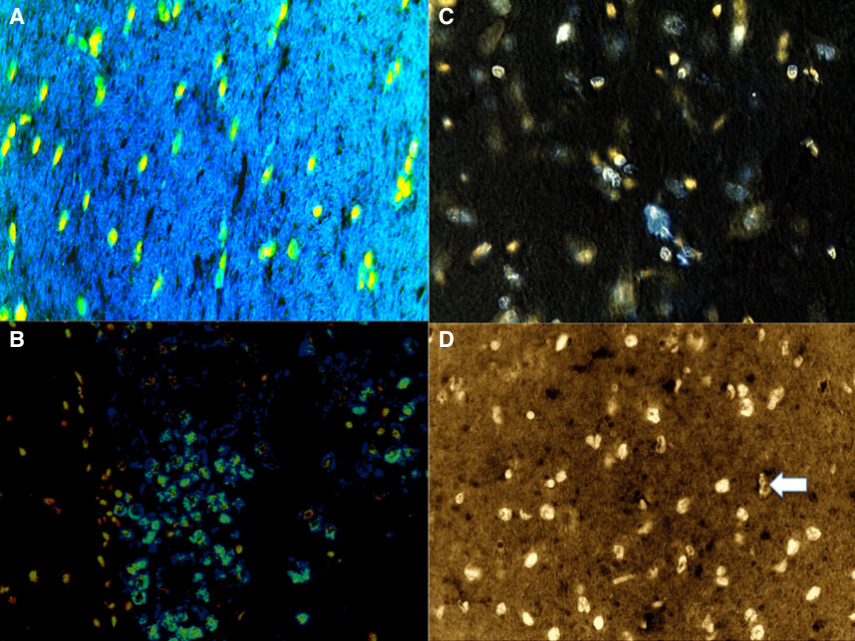
The neuronal nuclei labelled by TUNEL assay showed an intense, widespread, positive reaction in the treated group, especially in the frontal cortex: confocal laser scanning microscopy shows a marked positive nuclear reaction (in green apoptotic bodies) (**A**), and hippocampus (**B**) samples. Spotted positive nuclei were observed in cerebellum samples (in bleu) (**C**) and in the control group (arrow) (**D**).

The immunohistochemical study revealed an intensive positive result to TUNEL assay. The number of TUNEL positive cells that showed the typical morphological features of apoptosis (chromatin condensation, cytoplasmatic blebbing and apoptotic bodies) significantly increased in PFC, S and Hipp when compared with the control group. The neuronal nuclei labelled by TUNEL assay showed an intense, widespread, positive reaction in the treated group, especially in PFC, S and Hipp samples. Spotted positive nuclei were observed in cerebellum samples and in the control group (Fig. 6).

### Western blot analysis

Furthermore, the induction of expression levels was quantified by Western blot analysis. NF‐κB revealed an intense and massive positive reaction in ND group. Western blot analysis detected the chemiluminescent blots of NF‐κB, Bcl‐2 and SMAC/DIABLO in the treated group; a weak reaction was observed in the treated group for VMAT‐2.

Furthermore, the induction of these protein expression levels was quantified. The results were as follows: NF‐κB/β‐actin 0.60, Bcl‐2/β‐actin 0.60 and SMAC/DIABLO/β‐actin 0.50 for nandrolone‐treated group A; VMAT‐2/β‐actin 0.20 for the same ND group, matching perfectly with the immunohistochemistry results (Figs 2, 3, 4, 5, 6).

Control groups did not show any immunoreactivity for the studied markers, as well as Western blot analysis (reactions were not present), except for VMAT‐2. Our results are summarized in Table 2.

## Discussion

In this study, we have investigated the hypothesis that high, chronically administered doses of ND could induce deleterious effects in the brains of rats through a strict mutual crosstalk among apoptotic pathways activation, neuronal degeneration and oxidative stress unbalance. The results of the present study indicate that a long‐term administration of ND, an AAS, promotes oxidative injury in rat brain specific areas. The main metabolites are 3α‐hydroxy‐5β‐estran‐17‐one (3‐norandrosterone) and 3α‐hydroxy‐5β‐estran‐17‐one (2‐noretiocholanolone) 35. The substitution of a methyl group to the carbon atom at position 19 by a hydrogen atom in the testosterone molecule changes considerably the ratio between anabolic and androgenic activities, increasing the concentration of the former compound 35. The genotoxic activity of steroids is also because of an indirect process that takes place in the redox cycle, as well as in the production of oxygen reactive types 36, 37. Thus, the metabolic activation of testosterone derivatives leads to the formation of free radicals and consequently to the induction of oxidative stress. The lipid peroxidation observed in all brain areas tested, as an index of neuronal oxidative injury, is the evidence of this effect. The possible consequence on behaviour, learning, memory and cognitive abilities must be considered.

Our data revealed a strong increase in apoptotic death in brain specimens of treated rats when compared to the control group. Not surprisingly, we found that apoptotic death as indicated by the number of TUNEL+ cells was mostly exacerbated in brain areas of treated rats where the greatest densities of androgen receptors (ARs) were found, namely the hippocampus and deep layers of cerebral cortex where ARs were also localized 38, 39, 40, 41. As expected fewer TUNEL‐positive apoptotic cells were observed in cerebellum samples (*P* < 0.05).

The mechanisms of the neuropathological effects of AASs have not yet been completely clarified and are still largely unexplored; however, evidence has shown the recurrence of increased neuronal susceptibility to apoptotic stimuli as a source of the neurodegenerative and neurotoxic potential of these compounds 1. It is well known that AASs can exert apoptotic stimuli in various tissues and organs 42, 43, and growing evidence is emerging that apoptotic mechanisms are also partly involved in AASs induced neurotoxicity. Anabolic androgenic steroids mechanisms are similar to the other steroid hormones. In particular they exert their effects by binding to ARs at cellular level, translocating to binding sites on chromatin, promoting gene transcription, stimulating the production of mRNA and ultimately increasing protein synthesis 42. This classic genomic model for steroid hormone action presumes that steroid hormones can freely cross the plasma membrane, enter the cytoplasm, and bind to and activate specific intracellular steroid receptor proteins 44. An apoptotic effect of high dosages of AASs acting on an AR‐mediated genomic pathway has been experimentally demonstrated in dopaminergic neurons (N 27 cells) expressing ARs 45. In this experimental model, androgens enter the cell, bind to the classical intracellular ARs and induce oxidative stress leading to mitochondrial dysfunction. Release of cytochrome c from the mitochondria activates the apoptotic caspase cascade. This effect has been abrogated by the AR antagonist flutamide 25, 45.

In addition to the classical intracellular AR *via*, AASs can exert an apoptotic effect also through a non‐genomic pathway, involving the rapid rise of intracellular calcium concentration ([Ca^2+^]i) 46. The rapidity of the calcium modulation response (from seconds to minutes) leads us to presume that the androgen must bind to some sort of receptor at the surface of the cell to achieve this result 44. Interestingly, not all cell types that demonstrate a rapid androgen response express the classic nuclear ARs or are blocked by ARs antagonists. Therefore, it is not yet known whether the receptor located at the cell surface is the classic intracellular AR coupled to other signal transduction machinery located in the membrane or a unique protein, capable of binding androgens and initiating signal transduction cascade 46. Effects of AASs on intracellular Ca^2+^ represent a classic ‘non‐genomic’ effect; Ca^2+^ oscillations are a key point in neuronal apoptosis 47, 48. High, *supra*‐pharmacological doses of testosterone for relatively short periods initiate an apoptotic programme in neuroblastoma cells through a rapid overactivation of intracellular Ca^2+^ signalling pathways 49. This rapid effect of testosterone on intracellular Ca^2+^ signalling in neurons occurs in the absence of ARs 33, 49. The apoptotic role of AASs is further supported by the study of Tugyan *et al*. 50 who, in an animal model (rats), demonstrated that ND caused a significant increase in apoptotic cells and a significant decrease in neuronal counting in the parietal cortex, prefrontal cortex and hippocampal regions of the brain. Neuronal death was induced in the cortical neuronal cultures obtained from rats using high doses of nandrolone. The glial component is important in AASs‐induced neurotoxicity: when ND was administered to mixed (neuronal and glial cells) cortical cell cultures, low doses of the drug were enough to initiate the apoptotic death programme. Similarly in cultures of pure neurons, this toxic effect was inhibited by ARs antagonist flutamide 25. Conclusively, ND appears to be more potent in neurotoxicity when the glial component is present in cell cultures, suggesting that androgen‐induced brain inflammation through the induction of NF‐κB 51 could synergize with androgen in reducing neuronal viability 25. These observations are consistent with our findings of NF‐κB immunoreactivity that showed a strong positive reaction in brain samples of the treated rats compared to control group, particularly in PFC, S and Hypp samples. The NF‐κB family is a family of transcription factors that are central, co‐ordinating regulators of immunity, inflammation, development, growth and cell survival. In non‐stimulated cells, most of the NF‐κB complexes lie latent in the cells' cytoplasm interacting with IkB family inhibitory proteins. A great number of stimuli, including pro‐inflammatory cytokines, bacterial products and stress, can activate NF‐κB from these inactive cytosolic pools. When adequate stimuli occur, the IkBs proteins are quickly phosphorylated by the activating IκB kinase complex. Phosphorylation of inhibitory IκB proteins initiates their ubiquitination and subsequent proteosomal degradation, followed by the release and nuclear translocation of active NF‐κB dimers to regulate expression of target genes, among which are the encoding numerous cytokines, adhesion molecules, growth factors, immune receptors and prosurvival anti‐apoptotic proteins 52, 53, 54, 55, 56, 57, 58, 59, 60. The NF‐κB system is widely expressed in the central nervous system (CNS). Damage‐associated molecular patterns, pathogen‐associated molecular patterns, cytokines, chemokines, neurotransmitters, neurotrophic factors and neurotoxins are known to stimulate NF‐κB activation in the CNS 61, and the IKK/NF‐κB signalling system is thought to be critically involved in the pathogenesis of various neurological diseases 56, 62, 63. One of the earliest recognized unconventional functions of the apoptotic apparatus is represented by the death‐receptor‐mediated activation of NFκB‐regulated inflammation 64. NF‐κB is actually regarded as the matchmaker between apoptosis and inflammation 65. Ligand‐bound death receptors, in particular TNFR1, can potentially trigger a wide range of cellular responses ranging from cell death, because of extrinsic apoptosis or regulated necrosis, to NF‐κB activation. Depending on the cell type and specific context, NF‐κB can transactivate genes with anti‐apoptotic functions, such as BCL‐2, or leading to the production of pro‐inflammatory mediators including tumour necrosis factor‐α and interferon‐γ 58. Many other components of the extrinsic apoptotic pathway, such as some caspases, are also involved in the inflammatory response. Nevertheless, the exact role of the IKK/NF‐κB system in CNS pathology is not yet fully understood, it is argued that because of its pro‐inflammatory function, NF‐κB activation is able to trigger neuronal dysfunction, ageing and cell death, thereby increasing the severity of many CNS diseases 62, 66, 67, 68. Although this aspect has not yet been investigated, it is tempting to speculate that the neurotoxic effect of high doses of AAS can be mediated also by an inflammatory response through the pro‐inflammatory activity of some components of the apoptotic machinery. A link between oxidative stress and NF‐κB signalling pathways is supported by our results. In addition to high levels of oxidative stress, we consistently observed a strong immunopositivity to NF‐κB. It has been argued that one of the pathways leading to the activation of NF‐κB could be under reactive oxygen species (ROS)‐mediated control. In fact, growing evidence suggests that, although in limited doses, endogenous ROS may play an activating role in NF‐κB signalling, above a certain threshold, they may negatively impact upon this signalling 96. Reactive oxygen species are thought to have an inhibitory effect on NF‐κB activity. However, a mutual crosstalk between ROS and NF‐κB exists and recent studies have shown that ROS activity is subject to negative feedback regulation by NF‐κB, and that this negative regulation of ROS is the means through which NF‐κB counters programmed cell 69.

Bcl2 family members regulate the mitochondrial pathway of apoptosis. They are either pro apoptotic (Bak or Bax) or anti‐apoptotic (Bcl2 or Bcl XL), both of which are essential for apoptosis driven by the mitochondrial pathway. These proteins play a role in the permeabilization of the mitochondrial outer membrane on receiving apoptotic signals. Permeabilization leads to the release of cytochrome c, formation of apoptosome complex, activation of caspases, thus triggering morphological changes like membrane blebbing and nuclear fragmentation. Cell survival or apoptosis relies on the delicate balance between the up‐ and down‐regulation of Bcl2 and Bax 70, 71. Bax up‐regulation leads to enhanced susceptibility to apoptosis; on the contrary, Bcl2 up‐regulation leads to neuronal survival 72.

Our findings that chronic exposure to ND can impact VMAT‐2 levels are of interest. In particular, exposure to ND induced a significant (*P* < 0.001) decrease in VMAT‐2 immunoreactivity as assessed in tissue samples prepared from rat brain. VMAT‐2 is an important regulator of intra‐neuronal monoamine concentrations and disposition. It has been shown to be responsible for sequestrating cytoplasmatic neurotransmitters such as dopamine (DA) within synaptic vesicles. Under physiological conditions, DA is largely confined to synaptic vesicles where it is protected from metabolic breakdown. Sequestration of DA into vesicles provides a protective environment against the intracellular production of ROS. In the cytoplasm, free DA can in fact give rise to the formation of cytotoxic free radicals. Oxidative metabolites of DA may conjugate with α‐synuclein to form an adduct of DA–α‐synuclein, which may stabilize the toxic form of α‐synuclein through a covalent bound to DA quinone 73, while also promoting selective neurotoxicity 74. Normally, the concentration of cytoplasmic DA is kept at a minimum by continuous pumping activity of VMAT‐2 75. Cytosolic DA increases levels of DA‐generated oxy radicals ultimately resulting in degeneration of DAergic neurons. Moving from the study of Cubells *et al*. 76, it has been argued that the redistribution of DA from a smaller environment inside synaptic vesicles to oxidizing environments outside vesicles favoured the formation of ROS within the DA neurons which contribute to DA loss 77. Therefore, we can say that a change in DA storage and release machinery is associated with DA neurons loss, probably because of a caspase‐independent ROS‐mediated apoptotic pathway 78.

VMAT‐2 is currently considered a marker of dopaminergic neurons integrity with neuroprotective function. Recently, its role in neurodegenerative disorders, such as PD, has been unravelled 79, 80, thus focusing on the fact that VMAT2 defects may be an early abnormality promoting mechanisms leading to nigrostriatal DA neuron death in PD. Studies have indicated that several exogenous substances influence VMAT‐2 81. In particular, psycho‐stimulants, both the releasers (*i.e*. amphetamine analogous) and uptake blockers (*i.e*. cocaine‐like drugs) interfere with the activity and sub‐cellular distribution of monoamine transporters (VMAT‐2 and DAT – dopamine transporter), and this mechanism is likely to be related to the neurotoxicity shown by these substances 81. Several investigators have assessed the impact of cocaine on cytoplasmic vesicles, wherein it was determined that cocaine administration increases DA transport into this cytoplasmic vesicular fraction 82. This effect was attributed to a redistribution of VMAT‐2 and associated vesicles from synaptosomal membranes into the cytoplasm 83, thus elucidating the mechanisms whereby cocaine alters DA signalling. Psycho‐stimulants like methamphetamine which act as releasers of DA by disrupting vesicular pH gradients and allowing vesicular DA to redistribute into the cytoplasm 75, 81, have been demonstrated to decrease striatal VMAT‐2 ligand binding 81. Administration of several other agents causing DA release decrease VMAT‐2 activity and/or immunoreactivity in a similar manner; these include a single administration of AMPH 81 and repeated injections of MDMA 84. These drugs can potentiate the oxidative mechanism of DA. VMAT‐2 is able to take up methamphetamine in monoaminergic vesicles, inducing the release of DA to the cytosol which is important for methamphetamine neurotoxicity. The role of cytosolic DA in methamphetamine neurotoxicity has been supported by the fact that the inhibition of DA synthesis protects against methamphetamine neurotoxicity while the inhibition of VMAT‐2 and monoamine oxidase exacerbate methamphetamine neurotoxicity 85.

On the basis of the data presented here and on our findings showing a significant decrease in VMAT 2 immunoreactivity in ND treated rats compared to control rats, we infer that ND chronically administered could induce alterations in the VMAT machinery and alter the VMAT‐2‐mediated DA uptake into monoaminergic vesicles, which is known to be an important neuroprotective mechanism in dopaminergic neurons.

Given previous experimental findings of ROS involvement in pathways leading to the activation of programmed cell death 86, 87, in our experiment, oxidative stress involvement was evaluated in different brain areas where apoptosis was detected and quantified. Our results clearly show that *supra*‐physiological doses of ND administered chronically are able to disrupt redox metabolism in the brain, characterizing an oxidative stress state in all the studied cerebral areas. The low significant statistical difference of oxidative stress marker (MDA) in cerebellum specimens of treated rats compared to control group may likely reflect the high percentage of cerebellar granule cells that have been demonstrated less vulnerable to oxidative stress‐induced cell death, *via* a mechanism involving an up‐regulation of the cellular antioxidant defence 88.

## Conclusions

**Figure 7:**
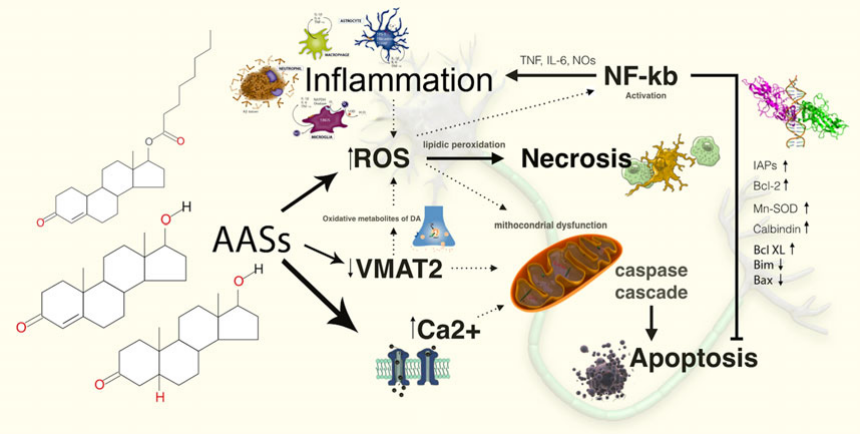
Mechanisms of the neuropathological effects of AASs: evidence has shown the recurrence of increased neuronal susceptibility to apoptotic stimuli as a source of the neurodegenerative and neurotoxic potential of these compounds. ROSs represent a serious hazard for cells, because they are powerful oxidizing molecules able to damage proteins, lipids and DNA. ROSs act as second messengers in various biological responses, among which the induction of programmed cell death is of paramount importance in our understanding of many common diseases and degenerative conditions. Growing evidence suggests that endogenous ROS may play an activating role in NF‐kB signalling, and above a certain threshold, they may negatively impact upon this signalling. ROS are thought to have an inhibitory effect on NF‐kB activity.

Thus, these findings support the idea that oxidative stress plays a pivotal role in AASs‐induced neurotoxiticy. ROS represent a serious hazard for cells because they are powerful oxidizing molecules able to damage proteins, lipids and DNA 89, 90. Reactive oxygen species act as second messengers in various biological responses, among which the induction of programmed cell death is of paramount importance in our understanding of many common diseases and degenerative conditions 91. Holmes *et al*. 92 investigated the effects of androgens under conditions of oxidative stress to determine whether androgens play a neuroprotective or neurotoxic role in DA neuronal functions. They found that androgens, alone, increased mitochondrial function *via* a calcium‐dependent mechanism. Androgen pre‐treatment protected cells from oxidative stress‐induced cell death. However, treatment with androgens after the oxidative insult increased cell death, and these effects were, in part, mediated by calcium influx into the mitochondria and the negative effects of androgens were not blocked by either androgen or oestrogen receptor antagonists 93. A membrane‐associated AR was thought to be implicated. The results of this study suggest that androgens are neuroprotective when oxidative stress levels are minimal, but when oxidative stress levels are elevated, androgens exacerbate oxidative stress damage 92. Similar results were reported by Cunningham *et al*. 94 who demonstrated that testosterone appears to have negative consequences on brain function under conditions of elevated oxidative stress. In a pre‐existing oxidative stress environment, androgens can further exacerbate oxidative stress damage 95, 96. A possible mechanism for androgen‐induced neuroprotection is preconditioning because androgens can moderately increase oxidative stress and apoptosis 25. These results suggest that the level of oxidative stress determines whether androgens play a positive or negative role in neuronal function 91, and it is argued that oxidative stress defines the neuroprotective and neurotoxic properties of androgens, thus acting as a molecular switch for androgen actions 94 (Fig. 7).

## Conflicts of interest

The authors confirm that there are no conflicts of interest.

## Author contribution

ET wrote the paper; MN performed the research; DC performed the research; SC contributed essential reagents or tools; PF performed the research; LM analysed the data; FPB analysed the data; CP analysed the data; IR performed the research; VF designed the research study and wrote the paper.
